# Circular RNA hsa_circ_0005046 and hsa_circ_0001791 May Become Diagnostic Biomarkers for Breast Cancer Early Detection

**DOI:** 10.1155/2021/2303946

**Published:** 2021-06-18

**Authors:** Melika Ameli-Mojarad, Mandana Ameli-Mojarad, Mitra Nourbakhsh, Ehsan Nazemalhosseini-Mojarad

**Affiliations:** ^1^Department of Biology Faculty of Basics Science, Kharrazi University Tehran, Tehran, Iran; ^2^Department of Biochemistry, Faculty of Medicine, Iran University of Medical Sciences, Tehran, Iran; ^3^Gastroenterology and Liver Diseases Research Center, Research Institute for Gastroenterology and Liver Diseases, Shahid Beheshti University of Medical Sciences, Tehran, Iran

## Abstract

Breast cancer (BC) is one of the most common lethal diseases in women worldwide. Recent evidence has shown that covalently closed Circular RNA (circRNA) deregulation is observed in different human malignancies and cancers. Lately, circRNAs are being considered as a new diagnostic biomarker; however, the mechanism and the correlation of action between circRNAs and BC are still unclear. In the present study, we try to investigate the expression level of hsa_circ_0005046 and hsa_circ_0001791 in BC. By using quantitative real-time polymerase chain reaction (qRT-PCR), expression profiles of candidate circRNAs were detected in 60 BC tissue and paired adjacent normal tissues. Furthermore, the clinicopathological relation and diagnostic value were estimated. Our results showed the higher expression levels of hsa_circ_0005046 and hsa_circ_0001791 in BC tissues compared to paired adjacent normal tissues with *P* value (*P* < 0.0001) for both circRNAs, and the area under the receiver operating characteristic (ROC) curve was 0.857 and 1.0, respectively; in addition, a total 10 miRNAs that can be targeted by each candidate circRNAs was predicted base on bioinformatics databases. Taken together, for the first time, the results of our study presented high expression levels of hsa_circ_0005046 and hsa_circ_00017916 in BC; although there was no direct correlation between the high expression level of both circRNAs with clinic pathological factors, except hsa_circ_0001791 association with estrogen receptors (ER), high ROC curve in expressed samples indicated that both circRNAs could be used as a new diagnostic biomarker for BC. Moreover, miRNAs selection tools predicted that miR-215 and mir-383-5p which have a tumor suppressor role in BC can be targeted by our candidate circRNAs to affect the PI3K/AKT pathway; in conclusion, further studies are required to validate the oncogene role of our candidate circRNAs through the PI3k pathway.

## 1. Introduction

Breast cancer (BC) is the most occurring cancer leading to death among women around the world, with more than two million new BC cases in females of all ages detected in 2020 [1].

It has been the most serious malignant tumor for women in developed and developing countries featured with the accumulation of mutations that result in the uncontrolled growth of BC tissues [2]. The major risk factors that influence information and development of BC include genetic risk factors, family history, female gender, age, alcohol, and race [3].

BC treatment is multidisciplinary, and despite surgical treatment and irradiation, chemotherapy remains one of the important means to treat BC; chemotherapy improved with combinations of adjuvant treatment such as taxane and a new targeted drug such as liposomal anthracyclines is among the most effective treatments for patients with BC [4, 5].

Although the diagnosis methods and therapy strategies for BC are developing, unfortunately, mortality from this cancer is still significant. Therefore, a better understanding of genetic changes in BC can help us to find related molecular mechanisms and genetic markers in prognosis and targeted therapy [6]. Recently, high-throughput RNA sequencing studies identified a large number of covalently closed Circular RNAs (circRNAs), a new class of noncoding RNAs, which generated from back splicing and could escape from exonuclease-mediated degradation and also more stable in blood or plasma compared to linear RNAs. This characterization has made circRNAs the best candidate for a new diagnostic or prognostic biomarker for cancers, such as BC [6, 7].

Previous studies have shown that there is a close correlation between circRNAs and initiation and progression of different cancers and diseases; however, knowledge about the correlation between noncoding RNAs, especially the circRNAs and BC, is not fully explored [7, 8].

Recently, abounded data show that circRNAs was found to regulate microRNA (miRNA) function by sponging miRNAs and play an important role in enhancing the expression levels of miRNA target genes; therefore, they can be used as a new gene expression regulator [8]. For example, circRNA named CDR1as, famed as ciRS-7, harboring more than 70 conserved binding sites and highly expressed in human and mouse brains was first reported to function as a sponge of miR-7 [9].

Tang et al.'s microarray showed that hsa_circ_00001982 expression is upregulated in BC tissues and, therefore, could be served as a diagnostic biomarker [10].

In this study, for the first time, we evaluated the expression level of hsa_circ_00005046 expression which is derived from the ARL8B gene (ADP-ribosylation-factor-like protein 8B) and circRNA ADAM9 hsa _circ_ 000179 which is derived from the ADAM9 GENE (disintegrin and metalloproteinase-domain-containing protein 9) [11]. In human BC tissues, compared to paired adjacent normal tissues, we determine a value of these circRNAs as a diagnostic or prognostic biomarker and investigate the correlation between expression levels of hsa_circ_0005046 and hsa _circ_ 000179 with clinicopathological data of BC patients.

## 2. Materials and Methods

### 2.1. Ethics, Consent, and Permissions

This study was approved by the Ethics Committee of the Rasoul Akram Hospital (code: IR.IUMS.FMD.REC.1398.218). BC patients were enrolled in the study and informed consent was obtained from all subjects, and none of the patients had previously undergone chemotherapy.

### 2.2. Patients and Tissue Specimen Collection

A total of 60 samples and paired adjacent normal tissues were obtained from patients during the surgery from Rasoul Akram Hospital between January 2017 and January 2018. All tumors were assessed by two experienced pathologists and frozen in liquid nitrogen after resection and stored at −80°C until required.

### 2.3. RNA Extraction

Total RNA was extracted from samples using the TRIZOL reagent (Sigma) according to the manufacturer's recommendations. Then, the extracted total RNA was quantified by a using a NanoDrop 2000 (Thermo Fisher, Waltham, MA).

### 2.4. Reverse Transcription-Polymerase Chain Reaction

**Figure 1:**
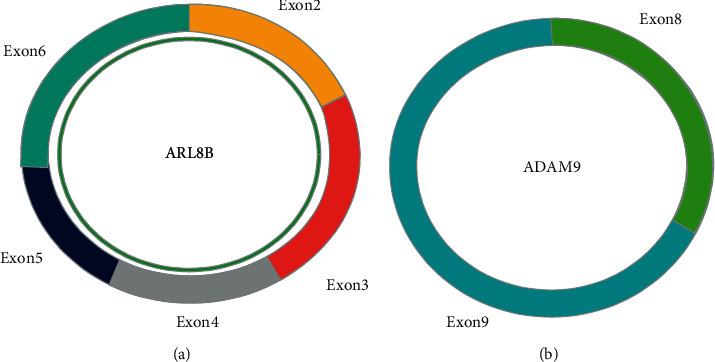
Schematic presentation of circular RNA (a) hsa_circ_0005046 generated from exon 2–6 of the *ARL8B* gene, containing locus, by a coding sequence (CDS) and (b) hsa_circ_0001791 generated from exon 8‐9 of ADAM9 gene locus by back splicing without CDS.

Total RNA was extracted from tissue as described above. The reverse transcription (RT) reaction of 10 mL total RNA was performed with random hexamer primers using a Revert Aid First Strand complementary DNA (cDNA) Synthesis Kit ABI (Applied Biosystems, CA) The polymerase chain reaction (PCR) amplification was performed using the ABI Prism7500 system (Applied Biosystems, CA), which is based on the SYBR green method. The cycling program is 95°C for 30 seconds, followed by 40 cycles of 95°C for 8 seconds and a preselected annealing temperature for 30 seconds. GAPDH acted as an internal control, and we performed qRT-PCR to measure differences between experimental batches. Then, the relative expression of genes was calculated using the 2^–ΔΔCt^ method. All the primers were designed using Circ primer software [12] and are shown in Table 1. hsa_circ_0005046 is generated from exon 2–6 of the ARL8B gene and hsa_circ_0001791 derived from the ADAM9 gene locus by back splicing (Figure 1).

### 2.5. Statistical Analysis

Statistical analyses were performed using SPSS 23.0 software (IBM, USA), Graph Pad Prism version 8.0 (Graph Pad Software, USA). Data were presented as mean ± SD. The fold change of each circRNAs was computed from the profile. The differences in levels of hsa_circ_00005046 and hsa_circ_0001791 between BC tissues and paired adjacent nontumorous tissues were assessed using the *t*-test for unpaired data; the receiver operation characteristic (ROC) curve was established to estimate the diagnostic values of hsa_circ_0005046 and hsa_circ_0001791 expression levels. The area under the ROC curve (AUC) was constructed as an accuracy criterion for examination of the hsa_circ_0005046 and hsa_circ_0001791 expression levels when the AUC was equal to 0.5, and the circRNAs was defined as having no diagnostic value.

### 2.6. Bioinformatics Analysis for miRNA Response Element (MRE)

Even though the functions of circRNAs have not been completely discovered, it is believed circRNAs can act as a “sponge” for miRNAs through their binding sites and modulate the activity of miRNA.

The CircInteractome (https://circinteractome.nia.nih.gov/index.html) bioinformatics database was used to predict the miRNAs that can be targeted by our selected circRNAs. Then, we filtered these miRNAs based on their involvement in the PI3K/AKT pathway by TargetScan subsequently (https://targetscan.org/vert_71/).

## 3. Results

### 3.1. CircRNAs Expression Profiles Revealed That hsa_circ_0005046 and hsa_circ_0001791 Were Upregulated in BC Tissue

**Figure 2:**
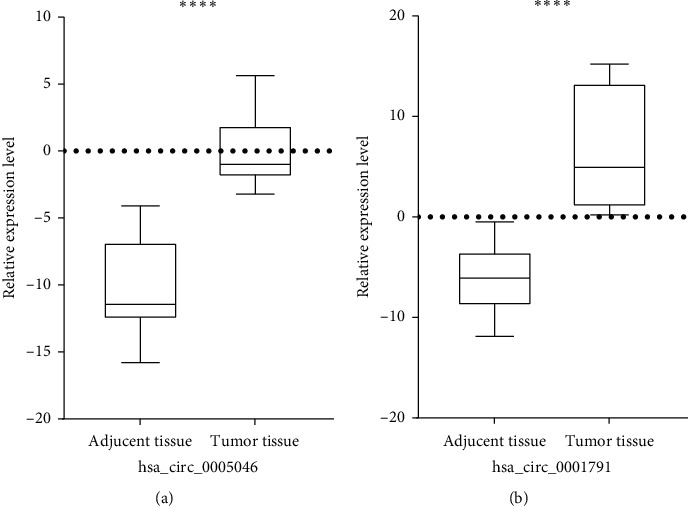
The relative expression levels of candidate circRNAs in BC tissues and control noncancerous tissues. (a) Illustrated photo of hsa_circ_0005046 and (b) illustrated photo of hsa_circ_0001791 both were overexpressed in BC tissues compared with adjacent normal tissues (^∗∗∗∗^*P* < 0.0001).

We collected 60 BC tissue samples and paired adjacent normal tissues using GAPDH as the internal control by qRT-PCR to examine the expression level of hsa_circ_0005046 and hsa_circ_0001791 in BC. The result showed that hsa_circ_0005046 and hsa_circ_0001791 expression in BC tissue was higher (*n* = 60, *P* < 0.0001) compared to those which are paired adjacent normal tissue (Figure 2).

### 3.2. Association between hsa_circ_0005046 and hsa_circ_0001791 Expression and Clinical Factors in BC

We analyzed the correlation between clinicopathological date and the expression level of hsa_circ_0005046 and hsa_circ_0001791. All clinicopathology data in the study are shown in Tables 2 and 3 including tumor size, TNM stage, age, lymph node status, distant metastases, and hormone receptors (HER2, ER, and PR).

60 patients with BC tumors were classified into two groups: a high-expression group and low-expression group according to the median expression value. We found that has_circ_0001791 expression was only associated with ER receptor (*P* value = 0.030), while hsa_circ_0005046 level does not correlate with age, gender, TMN stage, tumor grade, ER (estrogen receptor), PR (progesterone receptor), HER2, and lymphatic metastasis.

### 3.3. Potential Diagnostic Values of hsa_circ_0005046 and hsa_circ_0001791 in BC

**Figure 3:**
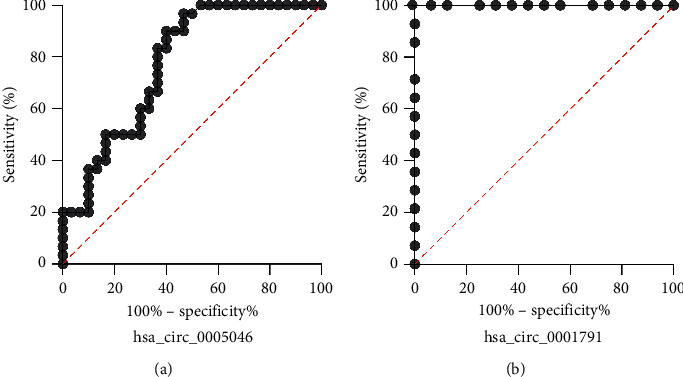
Potential diagnostic values of hsa_circ_0005046 and hsa_circ_0001791 in BC. (a) hsa_circ_0005046 (AUC) had 0.77 *P* value (<0.0001) (95% CI). The sensitivity and specificity were 0.85 and 0.51. (b) hsa_circ_0001791 (AUC) had 1.0 *P*value (<0.0001) (95% CI). The sensitivity and specificity were 0.1 and 0.87.

Besides the expression analysis, respectively, we estimated the potential diagnostic value of these molecules in BC by the ROC curve. The larger the area under the ROC curve (AUC), the higher the diagnostic value to determine the diagnostic values of circRNAs for cancer patients and ROC curve of hsa_circ_0005046 in BC patients. The area under the curve was 0.77 (*P*=0.02). The sensitivity and specificity were 0.85 and 0.51, respectively, as shown in (Figure 3).

### 3.4. Selected microRNA Result

The results obtained from the two mentioned databases are presented in Table 4. A total of 10 miRNAs were selected based on the number of binding sites.

## 4. Discussion

Over the last few decades, circRNAs have become a hotspot in the field of RNA as potential novel diagnostic and prognostic biomarkers, and more and more studies have investigated various diseases, such as cardiovascular diseases, and cancer research including BC [13, 14].

CircRNAs can also play a key role in a series of pathophysiology processes with the ability to affect gene expression and are closely associated with tumorigenesis, metastasis, and drug resistance [15].

In the present study, we investigate the high expression level of hsa_circ_0005046 and hsa_circ_0001791 by using qRT-PCR in 60 BC tissues compared to the paired adjacent tissues (*P* value = 0.0001), and ROC curve results indicated that the sensitivity and specificity of the assay were within the acceptable range (AUC = 0.77) for hsa_circ_0005046 and (AUC = 1.0) for hsa_circ_0001791, so they might be considered as new biomarkers.

A similar study by Xu et al. indicated that hsa_circ_0005230 was overexpressed in BC tissue and cell lines. Also, the overexpression is related to adverse phenotypes in the patients with BC. They proved the overexpression of this hsa_circ_0005230 decreased the expression of two miRNA miR-618 and miR-618 by directly sponging them. Therefore, hsa_circ_0005230 could be used as a prognostic predictor in BC patients [13].

Tang et al. proved hsa_circ_0001982 was significantly overexpressed in BC tissue. Also, knockdown of hsa_circ_0001982 suppressed BC invasion and cell proliferation and induced apoptosis by sponging miR‐143 [10].

More convincingly, circFBXW7 overexpression was found to inhibit cell migration and the proliferation of tumor growth both in vitro and in vivo. Also, circFBXW7 can suppress TNBC growth and metastasis with the ability to sponge omiR-197-3p and upregulating FBXW7 expression [14].

Although in our study, we could not find any significant correlation between the high expression of our candidate circRNAs and pathological features except circ0001791 association with ER, CircAGFG1 was discovered to be upregulated in TNBC (triple-negative breast cancer) tissues compared to adjacent normal tissues and the expression level of CircAGFG1 was correlated with the clinical stage, pathological grade, and poor prognosis of patients with TNBC [16].

Another study showed circANKS1B upregulation was correlated with TNM stage, distant metastasis, and histological grade, which can be applied in the staging and grading of BC [17].

Recently, the therapeutic potential of dysregulated circRNAs has been identified as a prognostic/diagnostic biomarker; for example, downregulated hsa_circ_0001785 in BC peripheral blood was found to be decreased in the plasma samples of postoperative BC patients compared to preoperative patients having the prognostic potential for BC treatment. Yin et al. also revealed that hsa_circ_0001785 in peripheral blood of BC patients was upregulated and might be a diagnostic biomarker with (AUC = 0.771, 95%CI: 0.592–0.915) for BC detection [18].

Similarly, circDENND4C is considered as a novel prognostic target for BC, since the high expression of circDENND4C was associated with tumor node metastasis stage and lymph node metastasis, and tumor size regulates proliferation under hypoxic condition [19].

**Figure 4:**
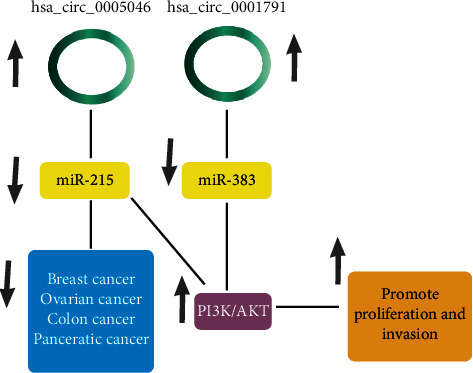
A schematic photo based on bioinformatics data prediction about the effect of upregulated circRNAs on their targeted miRNAs. hsa_circ_0005046 can target miR-215 and hsa_circ_0001791 can target miR-383, to promote proliferation and invasion in different cancers through the PI3K/AKT pathway.

Furthermore, we used bioinformatics databases to predicted miRNA that can be sponged by hsa_circ_0005046 and hsa_circ_0001791, since some specific circRNAs can target miRNA and negatively regulate miRNAs. Circ-ITCH is one of the well-known circRNAs with the ability to sponge miR-7, miR-17, and miR-214, and circ-ITCH can also upregulate the level of ITCH and the Wnt/*β*-catenin pathway [20]. Considering this study, miRNAs that can be targeted by hsa_circ_0005046 and hsa_circ_0001791 were selected by the CircInteractome and TargetScan database to specify the common miRNAs in PI3k/AKT signaling pathway. The results showed, mechanically, circ_0005046 acts as a miRNA sponge to directly inhibit hsa-miR-215 and circ_0001791 acts as a miRNA sponge to directly inhibit hsa-miR-383. MiR-215 is one of the downregulated microRNAs with a tumor suppressor role. Restoration of miR-215 expression inhibited the proliferation and invasion of BC and may be related to carcinogenesis and progression [21]. A study showed AKT serine/threonine kinase 1 (AKT1), a crucial factor of the PI3K/AKT pathway, was validated as a novel direct target of miR-215 and regulates various cellular processes, including, apoptosis, proliferation, migration, invasion, and metabolism of BC [21]. MiR-383-5p expression was downregulated in BC tissues and cell lines and has been characterized as a cancer suppressor in several cancers. The expression of miR-383-5p has an inhibitory effect on cell proliferation and was associated with differentiation, lymph node metastasis, and TNM stage. Results proved that miR-383-5p might have a tumor-suppressive effect by inhibiting the PI3K/AKT pathway [22]. A summary of the role of MiR-383-5p and miR-215 is illustrated in (Figure 4).

CircRNA specific structures with higher stability make them distinguished over linear RNAs. They can be also found not only in tissues but also in many human fluids, such as saliva and plasma, which make them potential to become a novel biomarker in cancer research. Despite a recent knowledge about circRNAs and their role to target miRNAs to regulate the expression involved in different important cellular pathways, including the cell cycle, migration, and apoptosis, their functions are not fully understood, and most studies are limited to tissues; the presence of circRNAs in bodily fluids needs to be investigated further to examine their use as diagnostic/prognostic targets in BC [5–23].

Our hypothesis from this study is that the upregulation of circ_0005046 and circ_0001791 may play important role in the downregulation of miR-215 and miR-383-5p via inhibiting the PI3K/AKT/mTOR pathway in BC.

## 5. Conclusions

Taken together, the findings of the present study indicated that hsa_circ_0005046 and hsa_circ_0001791 had different expression levels between BC tissue compared to adjacent normal tissues, and there is no study to find the interaction of hsa_circ_0005046 and hsa_circ_0001791 in BC prevention, for better understanding the role of hsa_circ_0005046 and hsa_circ_0001791. Further studies with larger sample size are needed to understand their molecular pathogenesis and tumorigenesis, and the ability of them to be a novel biomarker for early detection has been suggested.

## Figures and Tables

**Table 1:** qRT-PCR primers for hsa_circ_0005046, hsa_circ _0001791, and GAPDH.

CircRNA	Primer type	5ʹ-3′	Amplicon size (bp)	T for PCR
*hsa _circ_*0005046	Forward	TGCCTTGGATGAGAAACAGC	100	55.9
	Reverse	TCCTCATGTTGAAGCCCACT		

*hsa_ circ _*0001791	Forward	GATGTGCTGGGGAACTTCGT	94	55.9
	Reverse	CAGCTAGTTCTTATGACATGATGGG		

*GAPDH*	Forward	CGCTCTCTGCTCCTCCTGTTC	226	60
	Reverse	ATCCGTTGACTCCGACCTTCAC		

**Table 2:** The correlation of circ005046 expression levels and clinicopathological features of the BC patients. *P* < 0.05 was considered statistically significant.

hsa_circ_0005046				
Feature	No: 60	Low	High	*P* value
*Age*				
≤50	30	18	12	0.5
>50	30	13	17	

*Family history*				
Yes	36	15	21	0.2
No	24	14	10	

*TNM staging*				
I-II	27	18	15	0.2
III-IV	33	17	10	

*Depth of invasion*				
T1-T2	29	13	16	0.4
T3-T4	31	14	17	

*Lymphoid node infiltrated*				
Yes	33	17	16	0.31
No	27	10	17	

PR+	22	8	14	0.551
PR−	38	18	20	

ER+	33	25	8	0.5
ER−	27	17	10	

HER2+	31	15	16	0.3
HER2−	29	11	18	

**Table 3:** The correlation of circ0001791 expression levels and clinicopathological features of the BC patients. *P* < 0.05 was considered statistically significant.

hsa_circ_0001791				
Feature	NO:60	Low	High	*P* value
*Age*				
≤50	28	18	20	0.75
>50	22	12	10	

*Family history*				
Yes	30	15	15	0.2
No	30	14	16	

*TNM staging*				
I-II	27	18	15	0.2
III-IV	33	17	10	

*Depth of invasion*				
T1-T2	29	13	16	0.4
T3-T4	31	14	17	

*Lymphoid node infiltrated*				
Yes	33	17	16	0.31
No	27	10	17	

PR+	22	8	14	0.551
PR−	38	18	20	

ER+	33	24	9	0.0306^*∗*^
ER−	27	16	11	

HER2+	31	15	16	0.3
HER2−	29	11	18	

**Table 4:** Prediction of targeted microRNAs for circRNAs by using CircInteractome.

Hsa_Circ-005046	Number of binding sites
hsa-miR-1179	1
hsa-miR-1184	1
hsa-miR-1205	1
hsa-miR-127-5p	1
hsa-miR-1305	1
hsa-miR-1324	1
hsa-miR-188-3p	1
hsa-miR-192	1
hsa-miR-215	1
hsa-miR-487a	1
hsa_Circ-0001791	Number of binding sites
hsa-miR-1236	1
hsa-miR-1244	1
hsa-miR-383	1
hsa-miR-587	1
hsa-miR-622	1
hsa-miR-634	1
hsa-miR-665	1
hsa-miR-766	1
hsa-miR-942	1
hsa-miR-143	

## Data Availability

The data that support the findings of this study are available from the corresponding author upon reasonable request.

## Abbreviations

BC: Breast cancer
ADAM9: Disintegrin and metalloproteinase domain-containing protein 9
ARL8B: ADP-ribosylation-factor-like protein 8B
AKT1: AKT serine/threonine kinase 1
TNBC: Triple-negative breast cancer.
